# Bilateral Phyllodes Giant Tumor. A Case Report Analyzed by Array-CGH

**DOI:** 10.3390/diagnostics10100825

**Published:** 2020-10-15

**Authors:** Francesco Fortarezza, Federica Pezzuto, Gerardo Cazzato, Clelia Punzo, Antonio d’Amati, Teresa Lettini, Mattia Gentile, Antonia Lucia Buonadonna, Marta Mariano, Angela Pezzolla, Gabriella Serio

**Affiliations:** 1 Department of Cardiac, Thoracic, Vascular Sciences and Public Health, Pathology Unit, University of Padova, 35121 Padova, Italy; francescofortarezza.md@gmail.com (F.F.); federica.pezzuto@phd.unipd.it (F.P.); 2 Department of Emergency and Organ Transplantation (DETO), Pathology Section, Breast Unit Care, University of Bari, Medical School, 70124 Bari, Italy; gerycazzato@hotmail.it (G.C.); damatiantonio@yahoo.it (A.d.); lettinit@yahoo.com (T.L.); marta.mariano05@gmail.com (M.M.); 3 Department of Emergency and Organ Transplantation (DETO), Surgery Section, Breast Unit Care, University of Bari, Medical School, 70124 Bari, Italy; clelia.punzo@uniba.it (C.P.); angela.pezzolla@uniba.it (A.P.); 4 Medical Genetics, “Di Venere” Hospital, 70131 Carbonara (Bari), Italy; mattiagentile@libero.it (M.G.); buonadonnal@libero.it (A.L.B.)

**Keywords:** breast tumor, phyllodes tumor, array-CGH

## Abstract

The breast phyllodes tumor is a biphasic tumor that accounts for less than of 1% of all breast neoplasms. It is classified as benign, borderline, or malignant, and can mimic benign masses. Some recurrent alterations have been identified. However, a precise molecular classification of these tumors has not yet been established. Herein, we describe a case of a 43-year-old woman that was admitted to the emergency room for a significant bleeding from the breast skin. A voluminous ulcerative mass of the left breast and multiple nodules with micro-calcifications on the right side were detected at a physical examination. A left total mastectomy and a nodulectomy of the right breast was performed. The histological diagnosis of the surgical specimens reported a bilateral giant phyllodes tumor, showing malignant features on the left and borderline characteristics associated with a fibroadenoma on the right. A further molecular analysis was carried out by an array-Comparative Genomic Hybridization (CGH) to characterize copy-number alterations. Many losses were detected in the malignant mass, involving several tumor suppressor genes. These findings could explain the malignant growth and the metastatic risk. In our study, genomic profiling by an array-CGH revealed a greater chromosomal instability in the borderline mass (40 total defects) than in the malignant (19 total defects) giant phyllodes tumor, reflecting the tumor heterogeneity. Should our results be confirmed with more sensitive and specific molecular tests (DNA sequencing and FISH analysis), they could allow a better selection of patients with adverse pathological features, thus optimizing and improving patient’s management.

## 1. Introduction

Breast cancer tumors have different morphological phenotypes and specific histopathological types, including a spectrum of rare breast tumors [1], with distinctive prognostic and clinical characteristics. Breast phyllodes tumor (PT) is a biphasic tumor that accounts for less than 1% of all breast neoplasms. It is characterized by a double component, composed of hypercellular stroma, and epithelial/myoepithelial lined spaced. Based on several morphological and stromal characteristics, (mitotic rate, stromal cellularity, stromal atypia, and infiltrative borders) PT is classified as benign, borderline, or malignant [2]. To date, a complete surgical resection with safe margins is the gold-standard treatment for all PTs, regardless of the classification. Borderline or malignant tumors may recur locally. Although only a low percentage of them metastasizes, most frequently to the lung, a close follow-up is required. Chemotherapy and radiotherapy do not significantly improve survival rates in metastatic tumors and are not currently recommended as a routine approach. The morphological grading of PT suffers from inter-observer variability even amongst experienced pathologists; this is mainly due to intra-tumoral heterogeneity and the lack of molecular markers predictive of recurrent risk or malignant transformation. A genetic risk has also been hypothesized for PT; indeed, cases have been described in patients with Li-Fraumeni syndrome, a rare condition caused by the p53 mutation [3,4]. Many PTs have been analyzed by an array-CGH: frequent chromosomal imbalances such as alterations of almost entire chromosomes 7 and 8, gains at 1q, 5p, and losses at 6q, 9p, 10p, and 13q were reported. Moreover, an increasing rate of genetic defects has been observed from benign to malignant tumors [5,6]. Alterations of *CDKN2A* (9p), *RB1* (13q), *TP53* (17p), *EGFR* (7p), *MED-12*, and *TERT* promoter genes are most frequently observed in borderline or malignant tumors but none of them have been assigned a prognostic role [7]. Yeong et al [8] reported that mutations in *PIK3CA*, *RB1, TP53, NF1, ERBB4,* and *EGFR* might promote progression of borderline to malignant phyllodes tumors. However, a precise molecular classification of these tumors has not yet been established.

## 2. Materials and Methods

### 2.1. Case Presentation

An uncommon case of bilateral giant PTs was diagnosed in a 43-year-old woman who was admitted to the breast surgery unit in March 2017 with pain and significant bleeding from her breast skin. There was no documented familial cancer history. Radiological examination was negative for axillary lymphadenopathies or metastasis to other sites. The patient underwent ample surgical excision of two masses; a nodulectomy on the right and a total mastectomy on the left side. 

The study was conducted in accordance with the Declaration of Helsinki and national and institutional standards. Written informed consent to the use of breast tissue for additional studies and for research purpose was obtained from the patient. Such consent has been designed in accordance with the internal policy approved by the ethical committee of University of Bari (approval code 679/RA, n° 1587/2017, 01/02/2017).

### 2.2. Histological and Immunohistochemical Findings

All biopsy specimens were fixed in 10% buffered neutral formalin and paraffin embedded. Histological sections were stained with hematoxylin-eosin. Mitotic count was performed on 10 high power field (HPF). The tumor grading was performed according to WHO breast tumor classification [2]. Immunohistochemical analyses were also carried out by using the following antibodies: vimentin (Novocastra, Leica biosystem, Buffalo Grove, IL, USA, clone V9, 1:100), CD10 (DAKO, Glostrup, Denmark, clone 56C6, prediluted), S-100 (Novocastra, Leica biosystem, IL, USA, clone EP32, 1:400), actin (DAKO, Glostrup, Denmark, clone 1A4, 1:100), desmin (Novocastra, Leica biosystem, IL, USA, clone DER11, 1:100), keratins 19 (DAKO, Glostrup, Denmark, clone RCK108, 1:200), MNF116 (DAKO, Glostrup, Denmark, clone MNF116, 1:50), 8.18 (DAKO, Glostrup, Denmark, clone 5D3, 1:100), estrogen (Novocastra, Leica biosystem, IL, USA, clone 6F11, 1:50), and progesterone (Novocastra, Leica biosystem, IL, USA, clone 16, 1:40) receptors.

### 2.3. CGH-Array Analysis

Genomic DNA was extracted from 5-µm sections of paraffin-embedded tissue with the Dneasy Tissue Kit (Qiagen, Hilden, Germany) according to the manufacturer’s instructions. Normal sex-matched DNA was extracted from peripheral blood lymphocytes according to standard hybridization procedures (Nucleon BACC3, Amersham Pharmacia Biotech, Bucks, UK). The Genome ARRAY slide microarrays (TechnoGenetics srl, Bouty, Italy) used in this study consisted of 3600 BAC clones, with a spatial resolution of approximately 1Mb, in known and fixed genomic positions. DNA labelling, hybridization on microarray, and slide washings were performed according to the manufacturer’s protocols.

## 3. Results

### 3.1. Macroscopic Examination

**Figure 1:**
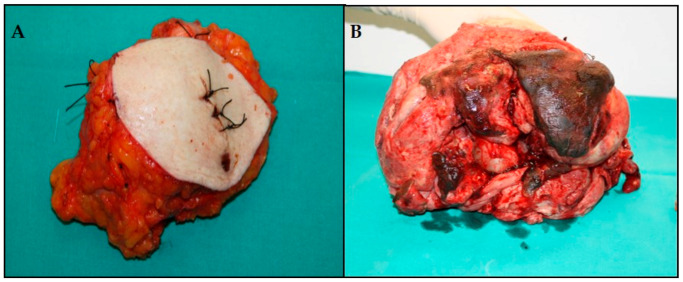
Macroscopic appearance of the bilateral breast PT: (**A**) The right nodulectomy; (**B**) The left mastectomy with necrosis and ulceration of the nipple and the skin.

On the right, a nodule measuring 12 cm in diameter was removed (Figure 1A). Macroscopically, the mastectomy specimen measured 22 × 18 × 14 cm and weighed 3750 gr, being almost completely occupied by a voluminous mass with extensive ulceration of the skin and the nipple (Figure 1B). 

### 3.2. Histological and Immunohistochemical Findings

**Figure 2:**
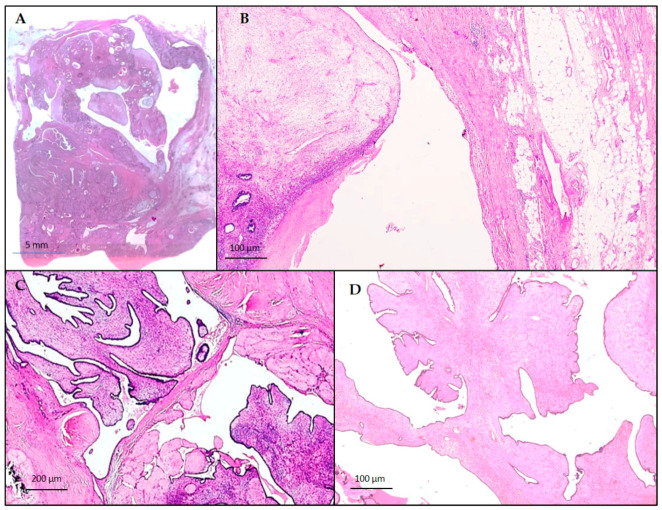
Borderline PT, appearing as a lobulated mass with focally permeating margins (**A**) hematoxylin and eosin, original panoramic view; moderately increased stromal cellularity (**B**) hematoxylin and eosin, original magnification × 50, and leaf-like epithelial pattern (**C**,**D**) hematoxylin and eosin, original magnification × 25, × 50.

On the right, histopathological examination described a sclerohyalin fibroadenoma within a spindle cell stromal proliferation, with well-defined borders, only focally permeative, a mitotic rate of 6/10HPF and a moderate increasing in stromal cellularity (Figure 2A–D). The diagnosis of a borderline phyllodes tumor associated with a sclerohyalin fibroadenoma was reported.

**Figure 3:**
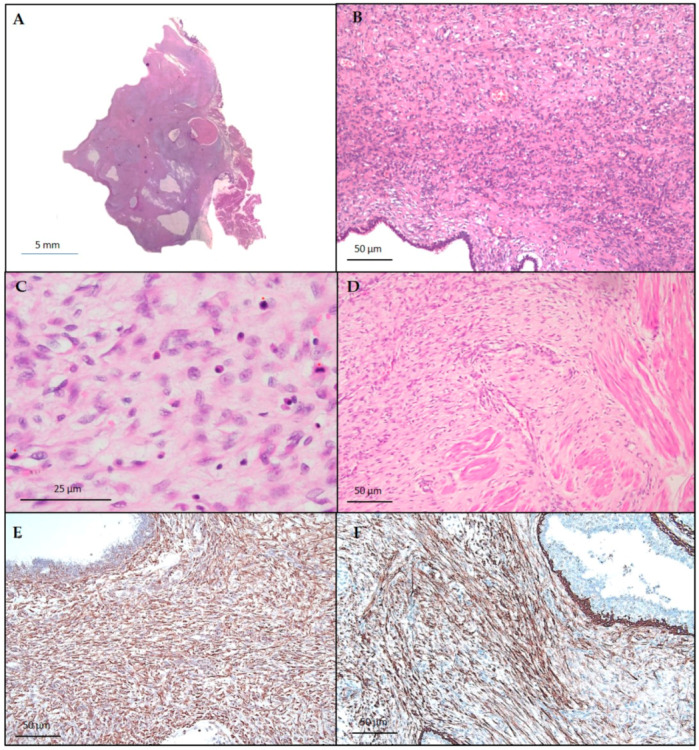
Malignant PT, showing marked and diffuse stromal cellularity and overgrowth (**A**) hematoxylin and eosin, panoramic view; (**B**) hematoxylin and eosin, original magnification × 100, pleomorphic stromal cells with brisk mitotic activity (**C**) hematoxylin and eosin, original magnification × 400, and permeative margins (**D**) hematoxylin and eosin, original magnification × 200. Immunohistochemical staining was positive for vimentin (**E**) original magnification × 100 and CD10 (**F**) original magnification × 100 in stromal cells.

Histopathological examination of the left tumor revealed a double-layered epithelial component surrounded by a stromal overgrowth, a periductular spindle cell proliferation with prevalent myxoid aspects, and a mitotic rate of more than 10/10 HPF (Figure 3A−D). The tumor border was permeative. No malignant heterologous component was found. The atypical stromal cells demonstrated positive immunoreactivity for vimentin and CD10 (Figure 3E,F), whereas reactions for S-100, actin, and desmin were negative. Keratins, estrogen, and progesterone receptors were also negative. The diagnosis was consistent with a malignant phyllodes tumor. 

### 3.3. CGH-Array Analysis

We analyzed the paraffin-embedded tumoral samples using an array-CGH to characterize copy number alterations that could be related to the tumorigenesis or clinical outcome. 

**Figure 4:**
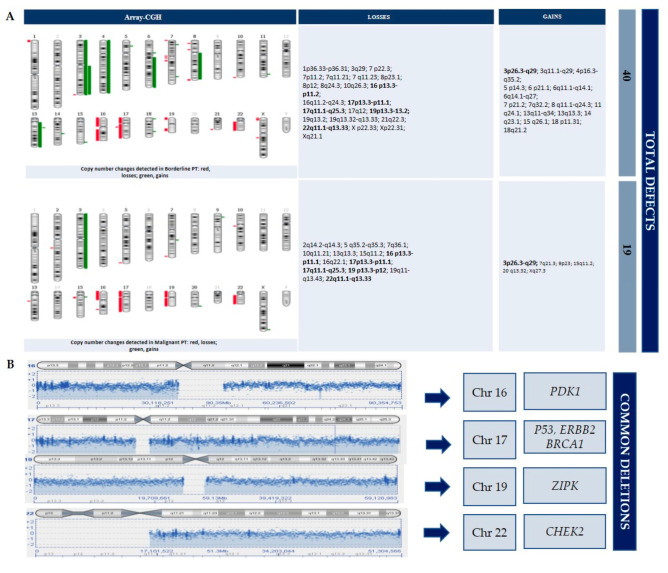
CGH-array results in borderline and malignant phyllodes tumor. (**A**) Shows all losses and gains in the borderline and malignant tumor, respectively. The alterations shared between the two entities are marked in bold. (**B**) The common deletions are listed.

DNA copy number changes were detected for each tumor (Figure 4). Losses were prevalent in both tumors. The borderline and malignant tumors showed identical loss regions at 16p13.3→p11.1, 17p13.3→p11.1, 17q11.1→q25.3, 19p13.3→p12, and 22q11.1→q13.33, as well as gains at 3p26.3→q29. Moreover, the borderline PT showed many total defects (40 total defects; 23 losses and 17 gains) and more than the malignant PT (19 total defects; 13 losses and 6 gains) (shown in Figure 2). Chromosome imbalances at 1q, 5p, 9p, and 10p, frequently reported in other studies, were not detected. No chromosomal defect was detected in the right fibroadenoma. At the last follow-up, 30 months after surgery, the patient was alive and disease-free. 

## 4. Discussion

Chromosomal instability is a major mechanism underlying genetic damage in cancer. Unlike other tumors, there is no evidence of the existence of a progression phase for different grades of phyllodes tumors. This has made it difficult to determine which risk factors and molecular changes could be responsible for the onset and progression of PT. A multi-step process is supported by the observation that numerous chromosomal deletions accumulate in most malignant tumors, many of which result in the loss and/or inactivation of TSGs. However, detecting the time scale of these genetic steps is difficult due to the rarity of malignant PT. It is not known whether it is a slow growing tumor after early genetic mutations, or the result of an accumulation of genetic changes reaching a threshold for malignant transformation. Chromosomal abnormalities increase with higher tumor grades, supporting the hypothesis that chromosomal instability is an early event in carcinogenesis. Using the whole-genome array-CGH strategy, we identified a high total number of chromosomal aberrations in both giant tumors described. Losses were more frequent than gains and many of these changes (losses and gains) overlapped. The identical minimally altered regions in our PTs were losses at 16p13.3→p11.1, 17p13.3→p11.1, 17q11.1→q25.3, 19p13.3→p12, and 22q11.1→q13.33, as well as gains at 3p26.3→q29. In this case, the recurrent chromosomal changes are largely consistent with previous genetic analyses performed in PTs [4,5,6,7]. Our study showed that gains were prevalent in the borderline tumor. This suggests that a higher number of gains could lead to the activation of either potent oncogenes or dominantly acting growth-regulating genes located in these regions. Considering this we aimed to discover which are the frequent and/or rare “driver mutations” in PTs. In fact, in a morphologically heterogeneous tumor, the identification of these alterations is essential to predict recurrences or metastasis, and so to implement personalized therapy. The chromosomal alterations shared between the borderline and the malignant tumor are hereby discussed. 

### 4.1. PIK3CA Gene

PI3K (phosphoinositide 3-kinase), the protein produced by the *PIK3CA* gene (3q26.32 locus), is part of a signaling pathway that has been extensively studied in the hope of halting the growth of metastatic breast cancer [6,8]. *PIK3CA* is part of the PI3K/AKT/mTOR pathway, a pathway that is involved in several different processes in cell growth. 

PI3K mutations are considered “driver mutations”, in which the proteins produced by the genetic changes drive the growth of the cell. *PIK3CA* mutations are thought to play a major role in breast cancer, including its development, evading cell death, the ability to spread and chemotherapy treatment resistance. Derangement of *PIK3CA* gene functions also appeared to be implicated in the progression of phyllodes tumors, together with the loss of p53 function [2,7,9,10]. In fact, 17p deletion (*TP53* gene) was one of the most common array-CGH changes observed in PTs and associated with malignancy; our tumors showed this defect, suggesting that *TP53* and *PIK3CA* are possibly early events inducing malignant transformation and growth. Although rarely, *PIK3CA* amplifications have been reported in some case series of invasive breast cancers [11,12]. However, somatic mutations rather than gains of gene copy number are the most frequently genetic alterations leading to human breast cancer progression. To the best of our knowledge, *PIK3CA* amplification has never been reported in phyllodes tumors. The finding of this rearrangement in our case is unique and needs a further large-scale comprehensive genetic study and a functional validation to understand its role in the biology of phyllodes tumors. 

### 4.2. PDK1 Gene

Pyruvate dehydrogenase kinase 1 (*PDK1*), the gene located at 16p13.3, is an isoenzyme that converts cytosolic pyruvate into the mitochondrial acetyl-CoA necessary for Kreb’s cycle. *PDK1* changes are reported in tumors such as lung, colon, melanoma, and breast. Although the mechanism is unclear, these would appear to be correlated with growth, migration, and metastatic capacity [13]. 

### 4.3. ZIPK Gene

The ZIP kinase (*ZIPK*) gene (19p13.3 locus) is a tumor suppressor gene (TSG) and a member of the death-associated-protein-kinase-family. Loss of apoptotic control reduces the sensitivity of tumor cells to programmed cell death and provides a powerful positive selection in tumor development. [14]. Loss at chromosome 19 seems to be important in tumorigenesis and progression in malignant PT [15]. 

### 4.4. BRCA1, CHEK2 and Other Tumor Suppressor Genes

Mutations of *BRCA1* (17q21.31 locus) are a rare event in phyllodes tumors [2]. This TSG is involved in the maintenance of DNA integrity; if not DNA-repaired, such breaks promote genomic instability and lead to the development of cancer. Other TSGs such as *MAPK1, TOP3B, PRAME, SOX 10*, and *CHEK2* are identified on chromosome 22 and some of them have been reported in breast cancers. The *CHEK2* gene (22q12.1 locus) encodes for checkpoint kinase 2 (CHK2), a protein that acts as a tumor suppressor. CHK2 regulates cell division and prevent cells from dividing too rapidly or in an uncontrolled manner [6]. When DNA undergoes a double-strand break, *CHK2* is activated. Specifically, the DNA damage-activated phosphatidylinositol kinase family protein (PIKK) ATM phosphorylates the Thr68 site and activates CHK2. Once activated, CHK2 phosphorylates downstream targets CDC25 phosphatases, which is responsible for dephosphorylating and activating the cyclin-dependent kinases (CDKs). Thus, CHK2 inhibition of the CDC25 phosphatases prevents cell mitosis. Furthermore, the CHK2 protein interacts with several other proteins including p53. Stabilization of p53 by CHK2 leads to cell cycle arrest in phase G1. The loss of normal CHK2 protein function leads to dysregulated cell division, accumulating DNA damage and, in many cases, tumor development. *CHEK2* mutations have been found to be associated with Li-Fraumeni syndrome-2 and seem to be associated with a higher contralateral breast cancer risk [2,15]. CHK2 interact with other genes such as *BRCA1/TP53/ATM*; the frequency is variable in different ethnic populations, highlighting a moderate/low breast cancer predisposition [2,16]. 

Furthermore, our case showed the loss of 17q12.21 locus, where *ERBB2* gene is located. *ERBB2* status in phyllodes tumor is unclear as well as the frequency of mutations [17]. Although *ERBB2* plays a main role in the classification and therapeutic management of breast cancer, its meaning in phyllode tumors is unknown. In two case series, the expression of c-erbB-2 was correlated with morphology and clinical outcome. c-erbB-2–positive tumors showed no particular histologic features [17] and did not differ between benign and malignant tumors [18]. Our finding was unexpected, and we are planning to perform further analyses on a higher number of cases to confirm these preliminary results.

## 5. Conclusions

Borderline tumors have an enigmatic behavior, an unclear pathogenesis, and controversial clinical management. Genomic instability is usually a hallmark of cancer, involved in its development and evolution. In our study, genomic profiling by assay-CGH revealed a greater chromosomal instability in the borderline mass (40 total defects) than in the malignant (19 total defects) giant phyllodes tumor. In accordance with previous studies, no chromosomal imbalances were found in the fibroadenoma. The larger number of losses present in the malignant tumor, a sign of the involvement of numerous TSGs, could be considered responsible for the malignant growth. The greater number of imbalances present in the borderline cancer could, on the one hand, be explained by tumor heterogeneity and, on the other, by a greater genetic instability. The common genetic imbalances observed in the different degrees of giant PT might be considered “drivers”, assisting pathologists in the morphological diagnosis and identifying those patients with different malignancy risks. These results need further confirmation with more sensitive and specific molecular analyses (DNA sequencing and FISH analysis), mainly focusing on coexisting masses of different histological grade as in our patient. This could identify possible alternative pathways of neoplastic progression, thus allowing a better selection of patients with adverse pathological features, optimizing patient’s management and improving clinical outcome.
